# MicroED Structures of Fluticasone Furoate and Fluticasone Propionate Provide New Insights into Their Function

**DOI:** 10.1021/acs.cgd.4c01683

**Published:** 2025-02-12

**Authors:** Jieye Lin, Johan Unge, Tamir Gonen

**Affiliations:** † Department of Biological Chemistry, University of California, Los Angeles, 615 Charles E. Young Drive South, Los Angeles, California 90095, United States; ‡ Department of Chemistry, Umeå University, 901 78 Umeå, Sweden; § Department of Physiology, University of California, Los Angeles, 615 Charles E. Young Drive South, Los Angeles, California 90095, United States; ∥ Howard Hughes Medical Institute, University of California, Los Angeles, Los Angeles, California 90095, United States

## Abstract

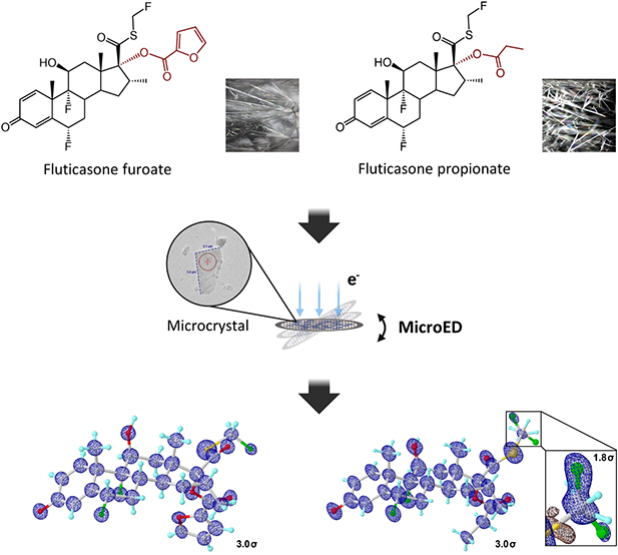

The detailed understanding of the conformational pathway of fluticasone, a widely prescribed medicine for allergic rhinitis, asthma, and chronic obstructive pulmonary disease (COPD), from formulation to its protein-bound state, has been limited due to a lack of access to its high-resolution structures. The three-dimensional (3D) structure of fluticasone furoate **1** remains unpublished, and the deposited structure of fluticasone propionate **2** could be further refined due to refinement against new data. We applied microcrystal electron diffraction (MicroED) to determine the 3D structures of **1** and **2** in their solid states. The preferred geometries in solution were predicted by using density functional theory (DFT) calculations. A comparative analysis of the structures of **1** and **2** across three states (in solid state, in solution, and protein-bound conformation) revealed the course of the conformational changes during the entire transition. Potential energy plots were calculated for the most dynamic bonds, uncovering their rotational barriers. This study underscores the combined use of MicroED and DFT calculations to provide a comprehensive understanding of conformational and energy changes during drug administration. The quantitative comparison also highlights the subtle structural differences that may lead to significant changes in the pharmaceutical properties.

## Abstract 2

The 3D structures of fluticasone furoate **1** and fluticasone propionate **2** in solid states were solved by microcrystal electron diffraction (MicroED). A comparative analysis of the structures of **1** and **2** across three states (in the solid state, in solution, and in the protein-bound conformation) revealed the conformational and energy changes during drug administration.

## 1 Introduction

Fluticasone is widely used to treat allergic rhinitis, asthma, and chronic obstructive pulmonary disease (COPD).^1−3^ In 2021, it was ranked as the 23rd most-prescribed medicine in the United States, with nearly 8 million patients and 25 million prescriptions per year.^4^ The common forms of fluticasone are fluticasone furoate **1** and fluticasone propionate **2**. Depending on the pharmaceutical formulation, they are marketed under different brand names, such as Flonase Sensimist,^5^ Arnuity Ellipta,^6^ Flovent Diskus,^7^ and Cutivate.^8^

Fluticasone is pharmaceutically formulated in three forms: nasal spray (in solution),^5^ powder-formulated inhaler (solid state)^6,7^ and ointment (in mineral oil).^8^ Characterizing the three-dimensional (3D) structures of **1** and **2** in their solid states has been of long-term interest, associated with the need for a low-energy conformation of the molecule as well as the understanding of the crystalline drug properties like solubility and stability. Previous research has shown that **1** could be cocrystallized with solvent molecules like dimethylformamide (DMF) and tetrahydrofuran (THF) (Table S1).^9−12^ In total, five of these structures were cocrystallized with their solvent molecules and were solved by single-crystal X-ray diffraction (SC-XRD). Those crystal forms are, however, not used in any pharmaceutical formulation, and moreover, none of them was disclosed to the scientific community. Most solvomorphs of **1** were microcrystals only, which could be indexed by powder X-ray diffraction (PXRD).^9−12^ The 3D structure of the unsolvated crystal form of **1** was therefore missing.^9−12^ As for **2**, two polymorph structures have been determined by SC-XRD and PXRD (Table S1).^13−15^ The SC-XRD structure (CSD entry: DAXYUX)^14^ showed disorder in the 17β-fluoromethylthioester moiety and a relatively higher R1 value (7.5%) as compared to the typical values in the range ∼4–6% for SC-XRD,^16^ prompting a reviewed model against additional new data; the other PXRD structure (CSD entry: DAXYUX01) was lacking the position of the hydrogen atoms.^15^

The development of the microcrystal electron diffraction (MicroED) technique bypassed the crystal size limitation for SC-XRD and is particularly suitable for micro- or nanosized crystals, i.e., crystals that are only a billionth of the size commonly used in SC-XRD.^17,18^ The models resulting from MicroED measurements are of comparable quality to SC-XRD structure determinations despite the need for microcrystals only. MicroED is particularly useful for determining the positions of bonded hydrogens, as a result of the relatively higher sensitivity of the electron beam to lighter atoms than X-rays.^19^ In this study, we applied MicroED to reveal the previously unavailable crystal structures of **1** and provide a refined structure of **2** in the solid state without any solvent molecule, with MicroED typical R-values.

Chemically, both **1** and **2** exhibit a consistent steroidal backbone with identical substitution groups; the only difference is the 17α-esterification, with a furoate ester in **1** and a propionate ester in **2**. This similarity in chemistry allows **1** and **2** to perform the same biological function, targeting the glucocorticoid receptor (GR) as agonists.^20,21^ Upon binding, the GR complex undergoes conformational changes and translocation into the nucleus, which can modulate gene expression.^22,23^ The binding affinity to GR differs between **1** and **2**, with **1** being nearly 70% stronger than **2**.^1,24^ In a study, **1** demonstrates a faster association rate and a slower dissociation rate compared to **2,** and the daily dose requirement for **1** is 110 μg, substantially less than the 200 μg dose required for **2**.^1,24^ The complex structures for GR/**1** and GR/**2** have been solved by X-ray crystallography and CryoEM (GR/**1**, PDB entries: 3CLD, 7PRV; GR/**2** was not disclosed).^20,21^ Structural analysis showed comparable residue interactions of GR/**1** and GR/**2**, but a better fit of the 17α-pocket for the furoate ester in **1** than the propionate ester **2**.^20^ The rationalization of the association and dissociation rate differences for **1** and **2** is not adequate without understanding their conformational and energy changes upon binding to the target protein in their protein-bound conformation. Smaller conformational changes may be related to a smaller energy barrier and lead to a faster association. Larger flexibility on the other hand, is related to an increase in entropy upon dissociation and a more favorable release. It may be speculated whether a lower energy barrier also facilitates the release of the compound from its binding position if there is a large difference between the bound and free form of the compound.

For both liquid- and powder-based formulations, drugs must dissolve before administration and functioning in the human body. The structure in solution therefore represents the intermediate conformation, the “transition state”, before the protein-bound conformation. However, a solvate structure is difficult to model in its equilibrium. In this study, we applied density functional theory (DFT) calculations to model solvent effects and predict the preferred geometries of **1** and **2** in water. Then, the three states of **1** and **2** (in the solid state, in solution, and in the protein-bound conformation) were compared to show the course of the conformational changes. Potential energy plots for the most dynamic bonds were calculated, examining their rotational barriers during the transition from the solid state to the protein-bound conformation, allowing us to quantitatively explain the structure–function differences between **1** and **2**.

## 2 Results and Discussion

**Figure 1:**
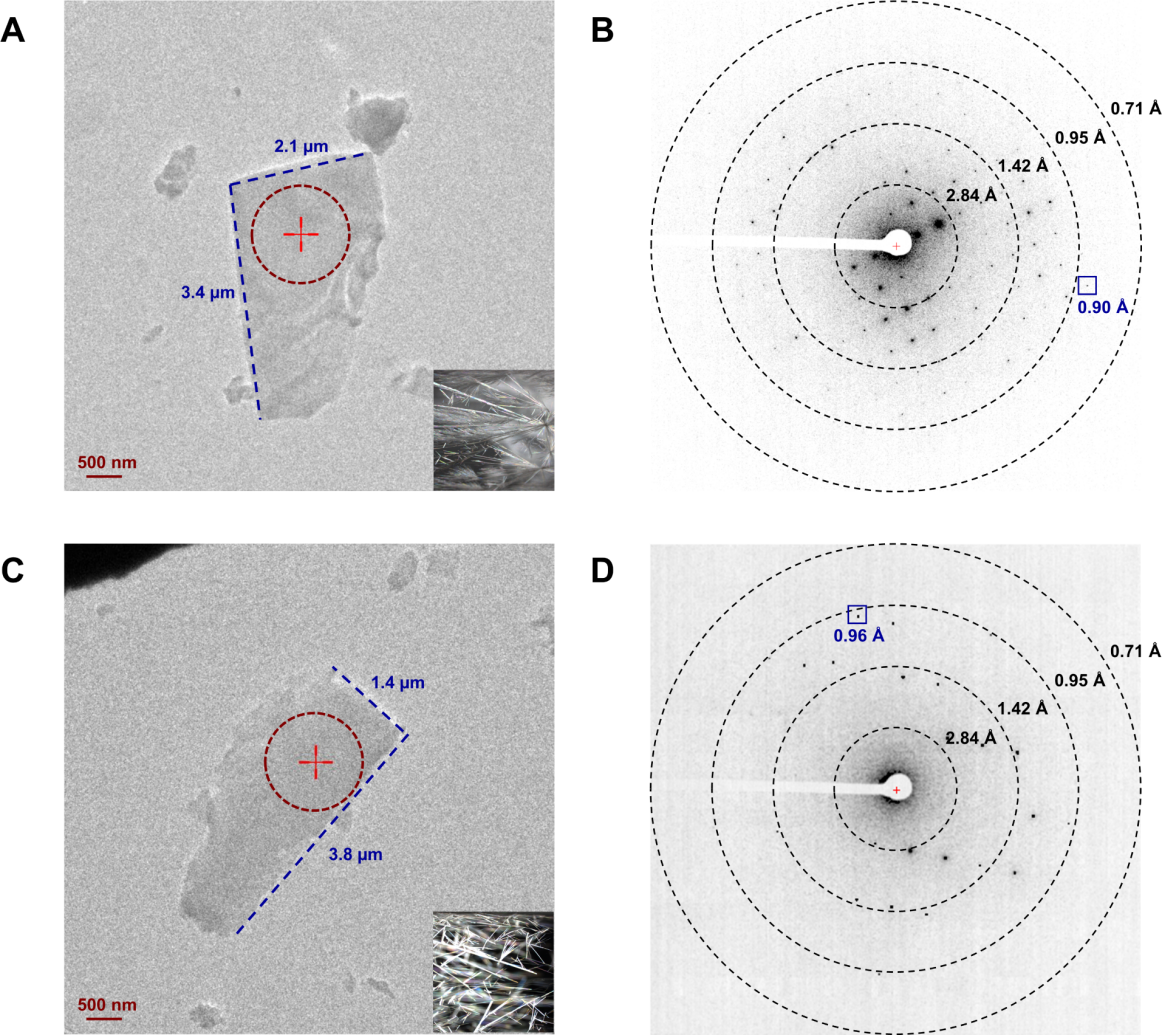
View of the crystals and their diffraction patterns. (A, C) Images of **1** and **2** under imaging mode (SA 5300×) and the stereo microscope (16×), respectively. (B, D) Diffraction patterns of **1** and **2** under diffraction mode (659 mm), respectively. The diffraction beam area was highlighted in dashed red circles in Figure 1A,C.

The commercially purchased **1** and **2** were recrystallized from methanol at room temperature, forming needle-shaped microcrystals on the surface of glass vials (Figure 1). Recrystallization was assumed to result in the unsolvated **1** and **2** following the procedure described.^11,12,25^ The crystals were gently ground into fine powders using a spatula. The MicroED grid preparation followed the procedure described in the literature (see “Methods” in the Supporting Information).^26^ The TEM grids containing microcrystals of **1** and **2** were loaded in a 200 keV Talos Arctica Cryo-TEM (Thermo Fisher). Crystals with a low contrast to the carbon support and therefore with an expected thickness of less than one micrometer were manually selected under the imaging mode (SA 3400×; see Figure 1) and calibrated to their eucentric heights to maintain them within the beam area during the continuous rotation. MicroED data were collected under diffraction mode with a camera length of 659 mm (the calibrated sample–detector distance). Typical data collection used a 0.5 s exposure time and 2° per second rotation rate settings over 120° wedges (−60° to +60°), which can be collected in ∼1 min with a total dose of ∼0.60 e^–1^/Å^2^ (electron dose rate: ∼0.01 e^–1^/(Å^2^·s)).^27^ The wide rotation wedge recorded high-tilt diffraction data to increase the completeness. To avoid diffraction overlap from nearby crystals or the grid bar, the starting and ending angles were manually examined and truncated.

**Figure 2:**
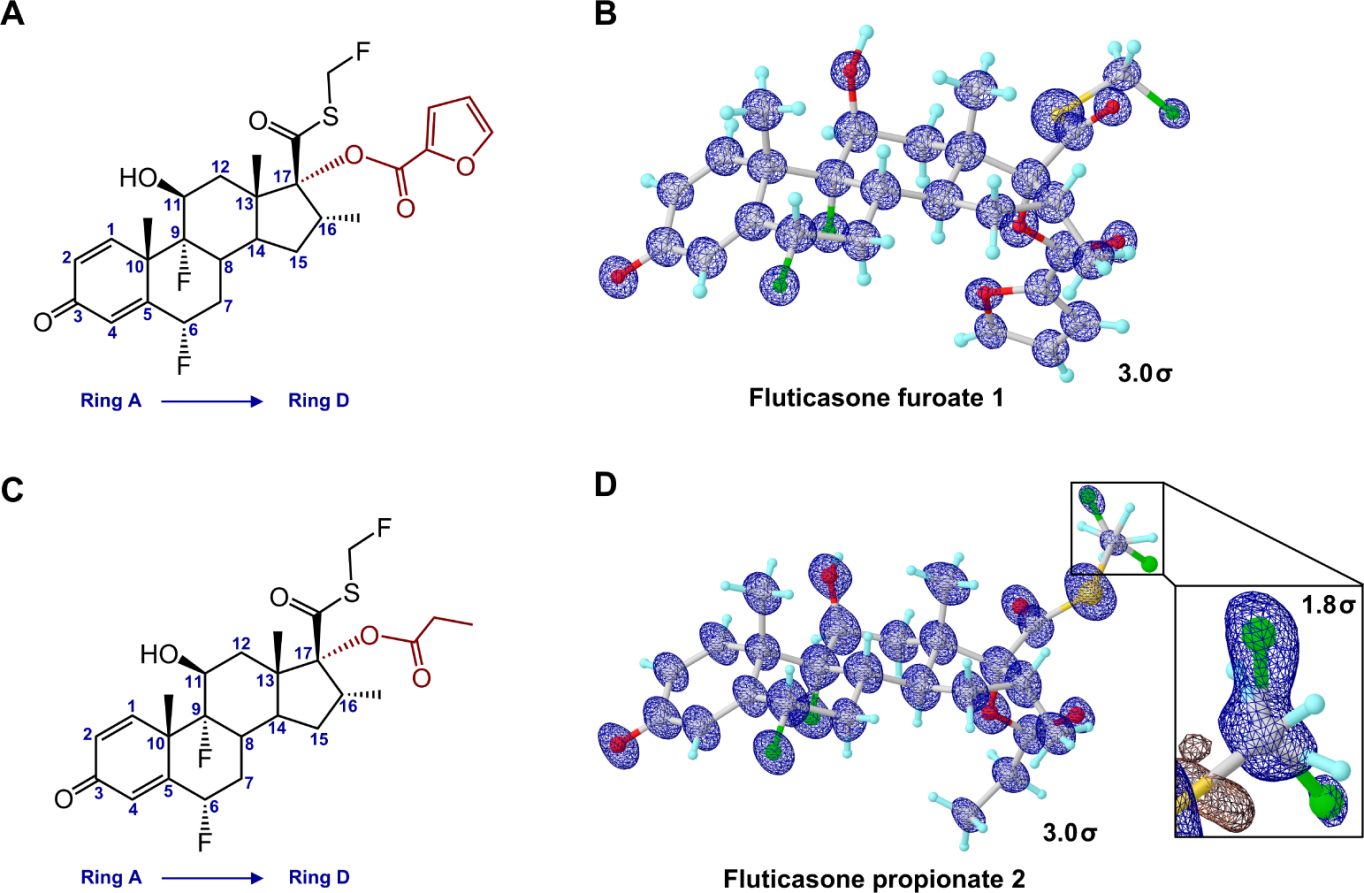
Chemical and MicroED structures. (A, B) Chemical structures of **1** and **2**, respectively. (C, D) MicroED structures of **1** and **2**, respectively. 2Fo-Fc density maps (3-sigma) were shown in blue mesh. The density of minor conformation of fluoromethyl group in **2** was shown in the expanded box at 1.8-sigma.

MicroED data were saved in MRC format and converted to SMV format using the mrc2smv software (https://cryoem.ucla.edu/microed).^27^ The converted frames were indexed, integrated, and scaled in XDS.^28,29^**1** was indexed with orthorhombic space group P 2_1_2_1_2_1_ (**a** = 7.70 Å, **b** = 13.95 Å, **c** = 23.48 Å, α = 90.0°, β = 90.0°, γ = 90.0°), and one data set alone was enough to reach ∼96% completeness; **2** was indexed with monoclinic space group P 2_1_ (**a** = 7.57 Å, **b** = 14.06 Å, **c** = 10.86 Å, α = 90.0°, β = 99.4°, γ = 90.0°), with four data sets merged to achieve ∼95% completeness (Table S2). The intensities were converted to SHELX HKL format using XDSCONV,^29^ and directly solved by SHELXD^30^ at the resolution of 0.90 and 0.96 Å for **1** and **2**, respectively. The structures were then refined by SHELXL,^31^ reaching the lowest R1 values of 16.4% and 15.3% for **1** and **2**, respectively (Table S2). Both R1 values of **1** and **2** fell in the typical range of ∼15–20% for MicroED structures, which are typically higher due to differences in the raw data, suggesting satisfying data qualities.^16^ The non-hydrogen atoms were accurately determined from the potential maps at subatomic resolution for **1** and **2** (Figure 2C, D). There is no evidence for additional atoms in the structures, and we draw the conclusion that both structures were unsolvated and devoid of methanol molecules. The 17β-fluoromethylthioester moiety in **1** showed no signs of disorder (Figure 2C), and the furoate ring oxygen atom (O6) was carefully examined by comparing the measurements of adjacent C–O (1.41 Å) and C=C (1.32 Å) bond lengths to their reference bond lengths.^32^ MicroED structure of **2** (Figure 2D) matched with the previously determined X-ray structure of **2** (RMSD: 0.05 Å; CSD entry: DAXYUX), which contained a disordered fluoromethyl group.^14^ The polar H atoms were located in the omit map, while the nonpolar H atoms were placed using riding models.^32^ Atoms were numbered following the steroid numbering convention described in the literature (Figures 2A,B and S1).^33^

**Figure 3:**
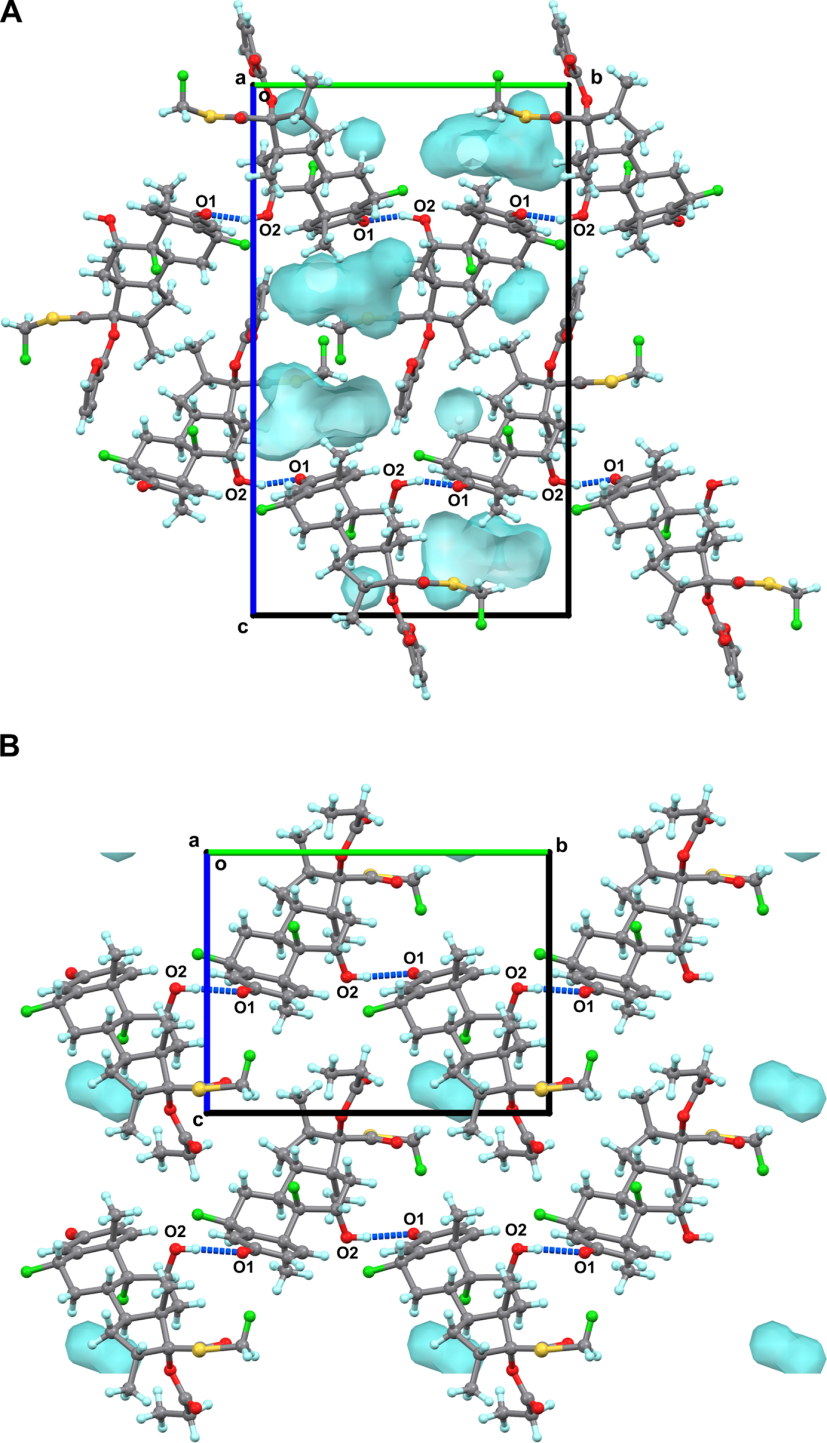
Packing diagram of **1** (A) and **2** (B), viewed along *a*-axis. Hydrogen bonding interactions were represented by the dashed lines in blue. Voids were detected by the same probe radius and grid spacing settings, colored in cyan. The minor conformation of **2** was omitted for clarification.

Crystal packing was compared for MicroED structures **1** and **2**. In **1**, molecules were tightly packed via a repetitive hydrogen bond O2–H**···**O1 (2.71 Å) between the 11β-hydroxy group (O2) and the 3-keto group (O1) along the *b*-axis (Figure 3A). A similar hydrogen bond O2–H**···**O1 (2.76 Å) was also found in **2** (Figure 3B). Weak contacts, such as C–H**···**F contacts (H**···**F < 3.0 Å),^34^ extend the packing along other directions but vary between **1** and **2**. For example, in **1**, C1–H**···**F2 (2.56 Å) and C25–H**···**F2 (2.93 Å) around the 6α-fluorine (F2) can extend crystal packing along *a*- and *c*-axes; while in **2**, three fluorine atoms (F1, F2, and F3A) form at least seven contacts that extend crystal packing along three axes, such as C19–H**···**F3A (3.39 Å), C24–H**···**F2 (3.41 Å), and C24–H**···**F3A (3.39 Å). The existence of the minor conformation of F3B bridges more contacts to C7 and C14. A nonuniform crystal growth with a preferred growth along the *b*-axis over the other two directions led to the plate- or needle-shaped morphologies in **1** and **2**. Voids in the unit cells of **1** and **2** were further examined, and it was found that 8.7% of the unit cell volume (55 Å^3^ per molecule) in **1** is accessible to solvent, whereas 0.8% of the unit cell volume (4.5 Å^3^ per molecule) is accessible to water in **2**, indicating a tighter packing in in **2** as compared to **1** and a better permeability of water in **1** than in **2.**

Examining the structural parameters for the asymmetric units of **1** and **2**, we found that both contain a rigid steroidal backbone composed of four fused rings (rings A–D, from left to right, see Figure 2A,B). These four rings act as conformational constraints for the overall backbone. Only a few differences were found in the steroidal backbone conformations of **1** and **2**. These minor variations occurred in the fusion bonds of rings B and C but did not affect rings A and D (Figure S1). The major structural differences between **1** and **2** were observed in the 17β-fluoromethylthioester substitution. For example, the C13–C17–C20–S1 is −74.2° in **1**, while it is 108.5° in **2** (Figure S1), with a nearly 180° difference. Rotation of the C17–C20 bond leads to more structural hindrances in the extended portion, with substantial rotational barriers. The 17α-esters in **1** and **2** share similar conformations but differ at the terminal side; for instance, the lipophilic part of the furoate ring (C25) positions outward due to the interaction with 6α-fluorine (F2) in **1**, whereas the flexible ethyl group (C24) positions backward to contact both 6α-fluorine (F2) and the 17β-fluoromethylthioester moiety (F3A) in **2** (Figure S1).

As the conformations of the two substitutions are different in **1** and **2**, as well as the rotational energy barriers required for the rotation of the substitutions in **1** and **2,** it seems likely that the free energy changes for **1** and **2** in their transition to the protein-bound conformation from the solid or liquid state are also different. These energy differences are also a result of the interactions within the active site in the protein, which depend on the exact chemical context and are not expected to be the same for the two molecules. This, in turn, influences the rate of transition from the dissolved state to the protein-bound conformation and significantly influences the pharmaceutical properties, like association/dissociation rate^35^ and biological half-life.^36^

**Figure 4:**
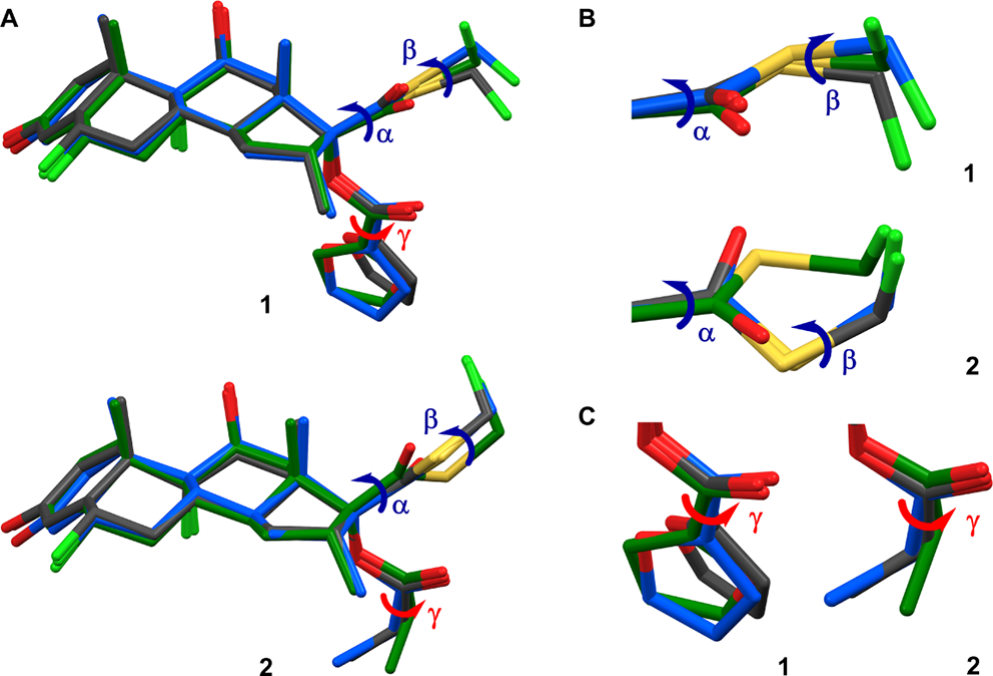
Structure overlays of **1** and **2.** (A) Overlay of the structures of **1** and **2** in the solid state (gray), in solution (blue), and in the protein-bound conformation (green). Three torsion angles, α (C13–C17–C20–S1), β (C20–S1–C21–F3), and γ (O4–C22–C23–O6 in **1**, and O4–C22–C23–C24 in **2**), were marked to represent the conformational changes from the solid state to the protein-bound conformation. A model was generated for **2** for the protein-bound conformation in agreement with images in the literature.^20^ The minor conformation of **2** in the solid state was omitted for clarification. (B) Expanded view and comparison of α and β torsion angles in **1** and **2**. (C) Expanded view and comparison of γ torsion angle in **1** and **2**.

Fluticasone is available as a nasal spray (in aqueous solution),^5^ inhaler (in solid state)^6,7^ and ointment (in mineral oil).^8^ The solid-state drugs need to be dissolved before interacting with the target protein; therefore, the solution structure represents a transition state prior to the protein-bound conformation. Modeling the structure in solution, however, is challenging since there is an ensemble of conformations at equilibrium. We applied density functional theory (DFT) calculations to model solvent effects and the preferred geometries of **1** and **2** in water (see “Methods” in the Supporting Information). Geometric optimization was performed using the functional/basis set combination B3LYP/6-31G(d,p),^37,38^ with the solvent effects of water modeled by the conductor-like polarizable continuum model (CPCM)^39^ and the solvation model based on density (SMD),^40^ both implemented in ORCA 5.0 software.^41^ The B3LYP/6-31G(d,p)^37,38^ optimized structures were further validated by comparing them with models calculated from ωB97*X*/6-311G(d,p)^42,43^ and B3LYP/6-311G(d,p)^38,43^ and showed no discernible variances caused by the different functional/basis sets. Comparing the structures of **1** and **2** in their solid states with predicted structures in solution showed minor conformational changes in the substitution groups (Figure 4). For example, in **1**, the O4–C22–C23–O6 has a 16° rotation, twisting the furoate ring from 15.4° to −0.6°; in **2**, the 3-keto group (O2) and propionate ester (C23, C24) exhibit at most a 0.4 Å movement due to molecular stretching. These minor conformational changes suggest that both **1** and **2** are conformationally constrained in the solid state and in solution, allowing them to maintain a constrained geometry during the transition to their protein-bound conformation in the protein pocket.

**Figure 5:**
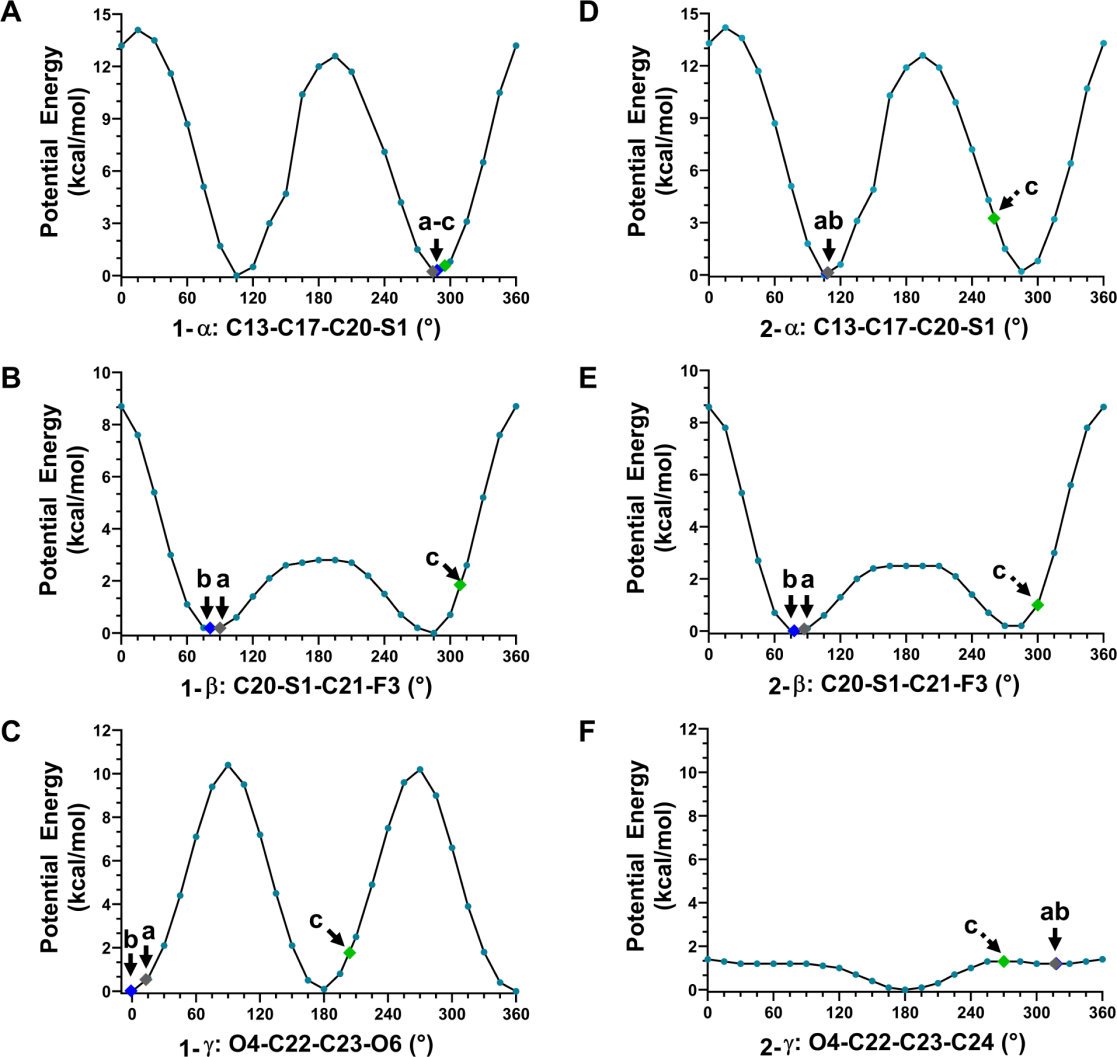
DFT-calculated potential energy plots for **1** (A–C) and **2** (D–F), showing the potential energy changes caused by a rotation of the corresponding torsions. Three points “a–c” were highlighted to represent the corresponding torsion angles measured from the solid state (point a), in solution (point b), and in the protein-bound conformation (point c).

Both **1** and **2** act as GR agonists in humans, and their complex structures have been reported (GR/**1**, PDB entries: 3CLD, 7PRV).^20,21^ GR/**2** was presented in the literature, but the structures were not disclosed; thus, a model was presented based on the figure in the literature for the purpose of comparison only (Figure 4).^20^ To understand the conformational changes when **1** and **2** undergo the transition from the solid state to the protein-bound conformation, the structures of **1** and **2** in three states (in the solid state, in solution, and in the protein-bound conformation) were compared (Figure 4). Three torsion angles α (C13–C17–C20–S1), β (C20–S1–C21–F3), and γ (O4–C22–C23–O6 in **1**, and O4–C22–C23–C24 in **2**) were identified as responsible for the major conformational changes. Notably, the β and γ torsion angles in **1**, and the α and β torsion angles in **2**, exhibited nearly 180° changes from the solid state to the protein-bound conformation (Figure 4B,C). To quantitatively model the rotational barrier and energy landscapes, the relative potential energy plots were calculated by scanning α, β, and γ torsions every 15° from 0° to 360° (see “Methods” in the Supporting Information). The values of α, β, and γ torsion angles and the corresponding potential energies in **1** and **2** were highlighted and compared (see points “a–c” in Figure 5).

The rotation of the α torsion angle significantly affects the overall conformation of the 17β-fluoromethylthioester moiety and involves a rotational barrier of ∼14 kcal/mol (Figure 5A and D). In the protein-bound conformation, the O3 atom of the carbonyl group is positioned outward, either hydrogen-bonded to the Cys736 residue (Figure S3B)^21^ or hydrophobically interacting with the Tyr735 residue (Figure S3A).^20^ Due to the large rotational barrier, it is energetically unfavorable to rotate the α torsion angle. In **1**, the α is relatively fixed, with 284° in the solid state, 288° in solution, and 295° in the protein bound conformation,^20^ resulting in an energy change of less than 1 kcal/mol (Figure 5A). On the contrary, **2** undergoes more than 150° rotation in α, with 109° in the solid state, 108° in solution, and ∼260° in the protein-bound conformation.^20^ Although **2** ends with a low energy value (∼3.3 kcal/mol), the transition still requires ∼13 kcal/mol to overcome the rotational barrier (Figure 5D). This may indicate a slower association rate with the receptor for **2** compared to **1**. Although the pathway is complex, it is more likely that the unbound 17β-fluoromethylthioester moiety in **2** is free to be metabolized to a 17β-carboxylic acid derivative with negligible glucocorticoid activity than **1** from this perspective.^1,2,44^

The rotation of the β torsion angle affects the position of the terminal fluorine atom (F3). In the complex structure GR/**1**, the F3 atom is found to be involved in a weak electrostatic interaction with the Asn564 residue (3.84 Å in 3CLD; 3.23 Å in 7PRV),^20,21^ as well as two hydrophobic interactions with the Phe749 and Thr739 residues (Figure S3).^20,21^ The C–F bond tends to be conformationally flexible, since a weaker density was experimentally detected in this region.^20^ Calculation of the potential energy plots for β in **1** and **2** showed a small rotational barrier (∼2.5 kcal/mol) ranging from 75° to 285° and a large rotational barrier (∼8.7 kcal/mol) in the remaining ranges (Figure 5B and E). The β in both **1** and **2** behaves flexibly and falls into that range; for example, in **1**, β is 90° in the solid state and 309° in the protein-bound conformation;^20^ in **2**, it is 87° in the solid state and ∼300° in the protein-bound conformation.^20^

Previous literature showed a better fit of the 17α-pocket for the furoate ring in GR/**1** than the propionate ester in GR/**2**, leading to different association and dissociation rates.^20^ Within the 17α-pocket, the furoate ring primarily hydrophobically interacted with the Met560, Leu563, Met639, and Met646 residues (Figure S3).^20,21^ This geometry resulted from the rotation of γ in **1**, i.e., γ is 13° in the solid state and ∼0° in solution, while it undergoes ∼190° rotations to 204° upon binding in the protein pocket. There is an ∼10 kcal/mol rotation barrier in the clockwise or anticlockwise direction, which is compensated by the above hydrophobic interactions (Figure 5C). A very weak hydrogen bond (3.93 Å) between the O6 atom of the furoate ring and the Gln642 residue may also be involved (Figure S3B).^21^ However, due to the low resolution reported in current X-ray (2.84 Å; PDB entry: 3CLD)^20^ and CryoEM (2.70 Å; PDB entry: 7PRV) structures,^21^ it cannot be verified whether the oxygen atom (O6) should be refined in its current position or the *meta*- position of the furoate ring, or if an alternative conformation with γ at 24° (180° flipping) coexists, since it maintains similar interactions but with minimal conformational changes (15° differences) and energy changes (less than 1.5 kcal/mol; Figure 5C). In contrast, the ethyl part of the 17α-propionate ester in **2** within the 17α-pocket is seen to be flexible and possesses a low rotational barrier (∼1.4 kcal/mol; Figure 5F). Regardless of the oxygen position mentioned above, the large rotational barrier of the furoate ring in **1** indicates that it is conformationally relatively rigid in the 17α-pocket and would be reluctant to dissociate; however, even though a smaller rotational barrier exists in **2,** there is an increase in entropy upon dissociation with a possible increased tendency toward release.

In this study, we utilized MicroED to determine the 3D structures of **1** and **2** directly from the microcrystals in powder, which could not be achieved by conventional structural characterization techniques. These structures reveal the differences in the conformations of **1** and **2** in their solid states. DFT calculations were employed to model the solvent effects and predict the preferred conformations of **1** and **2** in solution, representing the “transition state” before achieving their protein-bound conformations. Finally, we compared structures of **1** and **2** from the solid state, in solution, and their reported protein-bound conformations to identify the course of the conformational changes. It was found that the steroid backbones are extremely rigid for **1** and **2** in the entire pathway, while significant changes were observed on 17β- and 17α-substitutions, specifically in the α, β, and γ torsion angles (Figure 4). The potential energy plots for these torsion angles were calculated to estimate their energy landscapes, offering a quantitative approach to understanding their structure–function relationship. It was observed that the conformational changes of 17β-substitution in **2** require approximately 13 kcal/mol of energy to rotate the carbonyl group from a backward to an outward position, which is energetically unfavorable compared to less than 1 kcal/mol energy barriers in **1**, suggesting a faster association rate of **1** than **2** (Figure 5A and D). More unbound 17β-fluoromethylthioester moiety in **2** than **1** metabolized to 17β-carboxylic acid derivative results in a shorter biological half-life.^1,2,44^ While 17α-substitution cannot be metabolized by humans, after binding to the 17α-pocket, the furoate ring in **1** is more conformationally rigid (∼10 kcal/mol) than the propionate group in **2** (less than 1.4 kcal/mol), suggesting a decreased tendency for dissociation of **1** than **2** (Figure 5C,F). This study exemplifies the combined use of MicroED and DFT calculations to provide a comprehensive understanding of the conformational and energetic changes that may explain different pharmaceutical properties as the compounds undergo the changes from the solid state to their protein-bound conformation.

## Supporting Information Available

Materials, MicroED data collection, MicroED data processing, DFT calculations (see “Methods”); Literature summary (Table S1); MicroED data statistics (Table S2); Structural comparison (Figure S1); Models for DFT calculations (Figure S2); Diagrams of protein–ligand interactions (Figure S3) (PDF)
(PDF)

## References

[ref1] Biggadike K. Fluticasone furoate/fluticasone propionate–different drugs with different properties. Clin Respir J. 2011, 5 (3), 183 10.1111/j.1752-699X.2011.00244.x. 21569222 PMC3147057

[ref2] Fowler J.; Rotenberg B. W.; Sowerby L. J. The subtle nuances of intranasal corticosteroids. J. Otolaryng. Head Neck Surg. 2021, 50 (1), 18 10.1186/s40463-020-00480-z. PMC7968222 33731223

[ref3] Sorbera L. A.; Serradell N.; Bolos J. Fluticasone furoate. Drugs Future 2007, 32 (1), 1 10.1358/dof.2007.032.01.1065739.

[ref4] Kane S. P. The top 300 of 2021, ClinCalc DrugStats database, version 21.1. ClinCalc: https://clincalc.com/DrugStats/Top300Drugs.aspx. (Accessed 2025 January 27).

[ref5] Hosseini S.; Alfaifi A.; Esmaeili A. R.; Edwards D.; Schuman T.; Longest W.; Hindle M.; Golshahi L. Effects of nasal anatomical characteristics and administration parameters on delivery of locally-acting drugs with suspension nasal sprays in adults. J. Aerosol Sci. 2023, 167, 106101 10.1016/j.jaerosci.2022.106101.

[ref6] Hamilton M.; Leggett R.; Pang C.; Charles S.; Gillett B.; Prime D. In vitro dosing performance of the ELLIPTA® dry powder inhaler using asthma and COPD patient inhalation profiles replicated with the electronic lung (eLung). J. Aerosol Med. Pulm. Drug Delivery 2015, 28 (6), 498–506. 10.1089/jamp.2015.1225. PMC4685503 26372465

[ref7] Backman R.; Baumgarten C.; Sharma R. K. Fluticasone propionate via Diskus inhaler at half the microgram dose of budesonide via Turbuhaler inhaler. Clin. Drug Invest. 2001, 21, 735–743. 10.2165/00044011-200121110-00001.

[ref8] Katchman S. D.; Del Monaco M.; Wu M.; Brown D.; Hsu-Wong S.; Uitto J. Topical glucocorticosteroids: assay of biological strengths in a novel transgenic mouse model. J. Invest. Dermatol. 1995, 4 (104), 690. 7503571

[ref9] Kovacsne-Mezei A.; Gabriel R.; Jegorov A. Polymorphs of Fluticasone Furoate and Processes for Preparation Thereof. US 20,100,240,629 A1, 2010.

[ref10] Biggadike K.; Coote S. J.; Craig A. S.; Jacewicz V. W.; Millan M. J.; Nice R. K.; Noga B. M.; Seager J. F.; Theophilus A. L.; Crowe D. M. Anti-inflammatory Androstane Derivative Compositions. US 6,777,399 B2, 2004.

[ref11] Biggadike K.; Coote S. J.; Craig A.; Jacewicz V.; Millan M. J.; Seager J. F.; Theophilus A. L. Anti-inflammatory Androstane Derivative Compositions. US 6,777,400 B2, 2004.

[ref12] Biggadike K.; Chetina O.; Coote S. J.; Craig A.; Jacewicz V.; Millan M. J.; Seager J. F.; Theophilus A. L. Anti-inflammatory Androstane Derivative Compositions. US 6,858,593 B2, 2005.

[ref13] Coote S. J.; Nice R. K.; Wipperman M. D. Process for the Production of Fluticasone Propionate, in Particular of Polymorphic Form 1. EP 1,474,436 B12009.

[ref14] Cejka J.; Kratochvil B.; Jegorov A. Crystal structure of fluticasone propionate, C25H31F3O5S. Z. Fur Krist. - New Cryst. Struct. 2005, 220 (2), 143–144. 10.1524/ncrs.2005.220.14.153.

[ref15] Kariuki B. M.; Psallidas K.; Harris K. D. M.; Johnston R. L.; Lancaster R. W.; Staniforth S. E.; Cooper S. M. Structure determination of a steroid directly from powder diffraction data. Chem. Commun. 1999, 17, 1677–1678. 10.1039/A904702F.

[ref16] Aragon M.; Bowman S. E. J.; Chen C.-H.; de la Cruz M. J.; Decato D. A.; Eng E. T.; Flatt K. M.; Gulati S.; Li Y.; Lomba C. J. Applying 3D ED/MicroED workflows toward the next frontiers. Acta Crystallogr., Sect. C: struct. Chem. 2024, 80, 179–189. 10.1107/S2053229624004078. 38712546 PMC11150879

[ref17] Shi D.; Nannenga B. L.; Iadanza M. G.; Gonen T. Three-dimensional electron crystallography of protein microcrystals. Elife 2013, 2, e01345 10.7554/eLife.01345. 24252878 PMC3831942

[ref18] Nannenga B. L.; Shi D.; Leslie A. G. W.; Gonen T. High-resolution structure determination by continuous-rotation data collection in MicroED. Nat. Methods 2014, 11 (9), 927–930. 10.1038/nmeth.3043. 25086503 PMC4149488

[ref19] Clabbers M. T. B.; Martynowycz M. W.; Hattne J.; Gonen T. Hydrogens and hydrogen-bond networks in macromolecular MicroED data. J. Struct. Biol.: x 2022, 6, 100078 10.1016/j.yjsbx.2022.100078. 36507068 PMC9731847

[ref20] Biggadike K.; Bledsoe R. K.; Hassell A. M.; Kirk B. E.; McLay I. M.; Shewchuk L. M.; Stewart E. L. X-ray crystal structure of the novel enhanced-affinity glucocorticoid agonist fluticasone furoate in the glucocorticoid receptor–ligand binding domain. J. Med. Chem. 2008, 51 (12), 3349–3352. 10.1021/jm800279t. 18522385

[ref21] Postel S.; Wissler L.; Johansson C. A.; Gunnarsson A.; Gordon E.; Collins B.; Castaldo M.; Köhler C.; Öling D.; Johansson P.; Fröderberg Roth L. Quaternary glucocorticoid receptor structure highlights allosteric interdomain communication. Nat. Struct. Mol. Biol. 2023, 30 (3), 286–295. 10.1038/s41594-022-00914-4. 36747092

[ref22] Usmani O. S.; Ito K.; Maneechotesuwan K.; Ito M.; Johnson M.; Barnes P. J.; Adcock I. M. Glucocorticoid receptor nuclear translocation in airway cells after inhaled combination therapy. Am. J. Respir. Crit. Care Med. 2005, 172 (6), 704–712. 10.1164/rccm.200408-1041OC. 15860753

[ref23] Mortaz E.; Rad M. V.; Johnson M.; Raats D.; Nijkamp F. P.; Folkerts G. Salmeterol with fluticasone enhances the suppression of IL-8 release and increases the translocation of glucocorticoid receptor by human neutrophils stimulated with cigarette smoke. J. Mol. Med. 2008, 86, 1045–1056. 10.1007/s00109-008-0360-0. 18600309 PMC2517086

[ref24] Valotis A.; Högger P. Human receptor kinetics and lung tissue retention of the enhanced-affinity glucocorticoid fluticasone furoate. Respir. Res. 2007, 8 (1), 54 10.1186/1465-9921-8-54. 17650349 PMC1950704

[ref25] Murnane D.; Marriott C.; Martin G. P. Crystallization and crystallinity of fluticasone propionate. Cryst. Growth Des. 2008, 8 (8), 2753–2764. 10.1021/cg700954t.

[ref26] Jones C. G.; Martynowycz M. W.; Hattne J.; Fulton T. J.; Stoltz B. M.; Rodriguez J. A.; Nelson H. M.; Gonen T. The CryoEM method MicroED as a powerful tool for small molecule structure determination. ACS Cent. Sci. 2018, 4 (11), 1587–1592. 10.1021/acscentsci.8b00760. 30555912 PMC6276044

[ref27] Hattne J.; Martynowycz M. W.; Penczek P. A.; Gonen T. MicroED with the Falcon III direct electron detector. IucrJ. 2019, 6 (5), 921–926. 10.1107/S2052252519010583. 31576224 PMC6760445

[ref28] Kabsch W. Xds. Acta Crystallogr., Sect. D: biol. Crystallogr. 2010, 66 (2), 125–132. 10.1107/S0907444909047337. 20124692 PMC2815665

[ref29] Kabsch W. Integration, scaling, space-group assignment and post-refinement. Acta Crystallogr., Sect. D: biol. Crystallogr. 2010, 66 (2), 133–144. 10.1107/S0907444909047374. 20124693 PMC2815666

[ref30] Schneider T. R.; Sheldrick G. M. Substructure solution with SHELXD. Acta Crystallogr., Sect. D: biol. Crystallogr. 2002, 58 (10), 1772–1779. 10.1107/S0907444902011678. 12351820

[ref31] Sheldrick G. M. Crystal structure refinement with SHELXL. Acta Crystallogr., Sect. C: struct. Chem. 2015, 71 (1), 3–8. 10.1107/S2053229614024218. 25567568 PMC4294323

[ref32] Orpen A. G.; Brammer L.; Allen F. H.; Kennard O.; Watson D. G.; Taylor R. Appendix A: Typical Interatomic Distances in Organic Compounds and Organometallic Compounds and Coordination Complexes of the d-and f-block metals. Struct. Correl. 1994, 752–858. 10.1002/9783527616091.app1.

[ref33] Buchwald P.; Bodor N. Soft glucocorticoid design: structural elements and physicochemical parameters determining receptor-binding affinity. Pharmazie 2004, 59 (5), 396–404. 15212309

[ref34] Shukla R.; Chopra D. Crystallographic and computational investigation of intermolecular interactions involving organic fluorine with relevance to the hybridization of the carbon atom. CrystEngcomm 2015, 17 (19), 3596–3609. 10.1039/C4CE02391A.

[ref35] Miller D. W.; Dill K. A. Ligand binding to proteins: the binding landscape model. Protein Sci. 1997, 6 (10), 2166–2179. 10.1002/pro.5560061011. 9336839 PMC2143563

[ref36] Copeland R. A. Conformational adaptation in drug–target interactions and residence time. Future Med. Chem. 2011, 3 (12), 1491–1501. 10.4155/fmc.11.112. 21882942

[ref37] Tirado-Rives J.; Jorgensen W. L. Performance of B3LYP density functional methods for a large set of organic molecules. J. Chem. Theory Comput. 2008, 4 (2), 297–306. 10.1021/ct700248k. 26620661

[ref38] Petersson A.; Bennett A.; Tensfeldt T. G.; Al-Laham M. A.; Shirley W. A.; Mantzaris J. A complete basis set model chemistry. I. The total energies of closed-shell atoms and hydrides of the first-row elements. J. Chem. Phys. 1988, 89 (4), 2193–2218. 10.1063/1.455064.

[ref39] Barone V.; Cossi M. Quantum calculation of molecular energies and energy gradients in solution by a conductor solvent model. J. Phys. Chem. A 1998, 102 (11), 1995–2001. 10.1021/jp9716997.

[ref40] Marenich A. V.; Cramer C. J.; Truhlar D. G. Universal solvation model based on solute electron density and on a continuum model of the solvent defined by the bulk dielectric constant and atomic surface tensions. J. Phys. Chem. B 2009, 113 (18), 6378–6396. 10.1021/jp810292n. 19366259

[ref41] Neese F. Software update: The ORCA program system—Version 5.0. Wiley Interdiscip. Rev.: comput. Mol. Sci. 2022, 12 (5), e1606 10.1002/wcms.1606.

[ref42] Chai J.-D.; Head-Gordon M. Systematic optimization of long-range corrected hybrid density functionals. J. Chem. Phys. 2008, 128 (8), 084106 10.1063/1.2834918. 18315032

[ref43] McLean A. D.; Chandler G. S. Contracted Gaussian basis sets for molecular calculations. I. Second row atoms, Z= 11–18. J. Chem. Phys. 1980, 72 (10), 5639–5648. 10.1063/1.438980.

[ref44] Harding S. M. The human pharmacology of fluticasone propionate. Respir. Med. 1990, 84, 25–29. 10.1016/S0954-6111(08)80004-2. 2287792

