# Supplementary material for: MicroED Structures of Fluticasone Furoate and Fluticasone Propionate Provide New Insights into Their Function
Source: Cryst Growth Des. 2025 Feb 12;25(5):1588–96. doi: 10.1021/acs.cgd.4c01683 (PMC11887049; doi:10.1021/acs.cgd.4c01683)
Supplement: Supplementary file 1 — cg4c01683_si_001.pdf [file cg4c01683_si_001.pdf]

Supporting Information

MicroED Structures of Fluticasone Furoate and Fluticasone Propionate Provide New Insights to Their Function

Jieye Lin[1], Johan Unge[2] and Tamir Gonen[1,3,4*]

[1] Department of Biological Chemistry, University of California, Los Angeles, 615 Charles E. Young Drive South, Los Angeles, California 90095, United States

[2] Department of Chemistry, Umeå University, 901 87 Umeå, Sweden

[3] Department of Physiology, University of California, Los Angeles, 615 Charles E. Young Drive South, Los Angeles, California 90095, United States

[4] Howard Hughes Medical Institute, University of California, Los Angeles, Los Angeles, California 90095, United States

[*] Corresponding Author T.G. tgonen@g.ucla.edu

Methods

Materials

Fluticasone furoate **1** was commercially purchased from InvivoChem, and fluticasone propionate **2** was commercially purchased from Thermo Scientific Chemicals. Around 8 mg powders of **1** or **2** were separately dissolved in 2 mL methanol. Solvate was allowed to evaporate slowly in room temperature. Needle-shaped microcrystals formed on the surfaces of glass vials and were gently ground to powders using a spatula.

Grid preparation

The continuous carbon-coated copper grids (400-mesh, 3.05 mm O.D., Ted Pella Inc.) were pretreated with 15 mA negative glow-discharge plasma for 30 s using PELCO easiGlow (Ted Pella Inc.). The glow-discharged grids were separately mixed with powdery compounds of **1** and **2** in scintillation vials. After 30 s gentle shaking, microcrystals were absorbed on the surface of grids. The grids were clipped outside using c-clips and autogrid rings (Thermo Fisher).

MicroED data collection

The autogrids were loaded in a 200 keV Talos Arctica Cryo-TEM (Thermo Fisher) which is equipped with a CMOS CetaD camera (4096 × 4096 pixels) and EPU-D software (Thermo Fisher).[1] The microcrystals were screened under the imaging mode (SA 3400× and 5300×). Since the thickness of samples seriously affects the resolution of electron diffraction, only thin crystals with a certain brightness contrast were selected. Their eucentric heights were carefully calibrated to maintain the crystals inside the beam area during the continuous rotation. The MicroED data was collected in the diffraction mode at the camera length of 659 mm (the calibrated sample-detector distance) and beam size 11 under the parallel beam condition (45.2% C2 intensity). The 70 μm C2 aperture and 50 μm selected area (SA) aperture were used to reduce the background noise, which resulted an approximately 1.4 μm width beam area (electron dose rate: ~0.01 $e^{-1}/(Å^2 \cdot s)$).[2] Typical data collection used a constant rotation rate of 2°/s over an angular wedge of 120° from -60° to +60°, with 0.5 s exposure time per frame (~1 min per dataset), that result a total dose of ~0.6 $e^{-1}/Å^2$ for each item.

MicroED data processing

MicroED data was saved in mrc format and converted to smv format using the mrc2smv software (https://cryoem.ucla.edu/microed).[1] The converted frames were indexed and integrated by XDS.[3,4] **1** was indexed with space group P $2_12_12_1$ and only one dataset reached ~96% completeness. **2** was indexed with space group P 2_1. The common completeness for one single dataset ranged from 61~83%, therefore four datasets were merged to reach ~95% completeness. The merged data was scaled using XSCALE,[4] and their intensities were converted to SHELX hkl format using XDSCONV.[4] Structures of **1** and **2** were *ab initio* solved by SHELXD[5] and refined by SHELXL[6] using Shelxle[7] as a graphical interference (Table S2).

Density functional theory (DFT) calculation: geometric optimization

DFT calculations were conducted in ORCA 5.0[8] software using functional/basis set combination B3LYP/6-31G(d,p)[9,10] for geometric optimization. The effects of solvent water were treated using the conductor-like polarizable continuum model (CPCM)[11] and the solvation model based on density (SMD)[12] implemented in ORCA 5.0. The input coordinates were extracted from MicroED structures of **1** and **2** (the minor conformation was omitted). The molecules were allowed to be freely optimized during the calculations. Geometric optimized structures were further compared with other functional/basis set combinations like ωB97X/6-311G(d,p),[13,14] B3LYP/311G(d,p)[9,14] and showed no discernible differences.

DFT calculation: potential energy calculation

Three sets of DFT calculation were performed for structure **1** and **2** to evaluate the potential energy changes per bond rotation (see α, β, γ in Figure S2). Take set1 of **1** as an example, α (C13–C17–C20–S1) was rotated from 0° to 360° with 15° increment to generate 25 structures. The coordinates were extracted to generate 25 different input files. α was then fixed while the remaining structure was allowed to freely optimize during the calculations. DFT calculations were conducted within ORCA 5.0,[8] using functional/basis set combination B3LYP/6-31G(d,p),[9,10] and CPCM[11] and SMD[12] models for solvent water effects. The potential energy (relative energy) values were calculated by using the single point energy to substrate the minimum energy. The potential energy versus torsion angle was plotted using GraphPad Prism 8 software (Figure 5).[15]

Table S1 Summary of unit cell parameters for literature-reported polymorphs (solvomorphs) of **1** and **2**.

**Solvomorphs of 1**				
**1•Guest molecule**	**Space group**	**Unit cell parameters**	**Method**	**Refs**
Methy lacetate	$P2_12_12_1$	a=12.1 Å, b=14.6 Å, c=16.3 Å, β=90°	PXRD	[16]
1,3-dimethylimidazolidinone (DMI)	C2	a=30.4 Å, b=7.5 Å, c=14.7 Å, β=105.6°	PXRD	[16]
(S)-2-Butanol	$P2_12_12_1$	a=12.4 Å, b=15.5 Å, c=15.5 Å, β=90°	SC-XRD	[16]
Ethanol	$P2_12_12_1$	a=12.2 Å, b=15.2 Å, c=15.5 Å, β=90°	PXRD	[17]
Propan-1-ol	$P2_12_12_1$	a=12.4 Å, b=15.4 Å, c=15.5 Å, β=90°	PXRD	[17]
Propan-2-ol	$P2_12_12_1$	a=12.3 Å, b=15.1 Å, c=15.7 Å, β=90°	PXRD	[17]
1,4-Dioxane	$P2_12_12_1$	a=12.5 Å, b=14.6 Å, c=16.1 Å, β=90°	PXRD	[17]
Ethyl formate	$P2_12_12_1$	a=12.0 Å, b=14.7 Å, c=16.2 Å, β=90°	PXRD	[17]
Acetic Acid	$P2_12_12_1$	a=11.9 Å, b=14.5 Å, c=16.1 Å, β=90°	PXRD	[17]
Acetone	$P2_12_12_1$	a=11.9 Å, b=14.7 Å, c=16.2 Å, β=90°	PXRD	[17]
Dimethylformamide	$P2_12_12_1$	a=12.1 Å, b=14.8 Å, c=16.2 Å, β=90°	SC-XRD	[17]
Dimethylacetamide	$P2_12_12_1$	a=12.2 Å, b=14.9 Å, c=16.6 Å, β=90°	PXRD	[17]
Methylethylketone	$P2_12_12_1$	a=12.0 Å, b=14.9 Å, c=16.3 Å, β=90°	PXRD	[17]
Tetrahydrofuran	$P2_12_12_1$	a=12.0 Å, b=14.6 Å, c=16.4 Å, β=90°	SC-XRD	[17]
N-Methyl-2-pyrrolidinone	$P2_12_12_1$	a=12.0 Å, b=14.9 Å, c=16.8 Å, β=90°	PXRD	[17]
Butan-1-ol	$P2_12_12_1$	a=12.5 Å, b=15.7 Å, c=15.4 Å, β=90°	PXRD	[17]
Methyl acetate	$P2_12_12_1$	a=12.1 Å, b=14.6 Å, c=16.3 Å, β=90°	PXRD	[17]
Toluene	$P2_12_12_1$	a=7.8 Å, b=13.7 Å, c=34.2 Å, β=90°	PXRD	[18]
m-xylene	$P2_12_12_1$	a=7.8 Å, b=13.8 Å, c=35.9 Å, β=90°	PXRD	[18]
Fluorobenzene	$P2_12_12_1$	a=7.7 Å, b=13.9 Å, c=38.7 Å, β=90°	PXRD	[18]
Ethylbenzene	$P2_12_12_1$	a=7.7 Å, b=13.8 Å, c=37.8 Å, β=90°	PXRD	[18]
Chlorobenzene	$P2_12_12_1$	a=7.8 Å, b=13.7 Å, c=39.3 Å, β=90°	PXRD	[18]
Triethylamine	$P2_12_12_1$	a=7.5 Å, b=12.8 Å, c=34.4 Å, β=90°	SC-XRD	[19]
Diethylamine	$P2_12_12_1$	a=7.7 Å, b=12.7 Å, c=31.9 Å, β=90°	SC-XRD	[19]
Dipropylamine	$P2_12_12_1$	a=7.8 Å, b=13 Å, c=32.2 Å, β=90°	PXRD	[19]
**Polymorphs of 2**				
**2**	**Space group**	**Unit cell parameters**	**Method**	**Refs**
form 1	$P2_1$	a=7.7 Å, b=14.2 Å, c=11.3 Å, β=98.5°	PXRD	[20]
	$P2_1$	a=7.6 Å, b=14.1 Å, c=11.0 Å, β=99.3°	SC-XRD	[21]
form 2	$P2_12_12_1$	a=23.4 Å, b=14.0 Å, c=7.7 Å, β=90°	PXRD	[20]
	$P2_12_12_1$	a=23.2 Å, b=14.0 Å, c=7.7 Å, β=90°	PXRD	[22]

Notes: polymorphs without index of unit cell parameters were omitted for clarification.

Table S2 MicroED data statistics of **1** and **2**.

Compound	**1**	**2**
Name	fluticasone furoate	fluticasone propionate
Stoichiometric formula	$C_{27}H_{29}F_3O_6S$	$C_{25}H_{31}F_3O_5S$
Mr	538.56	500.56
Temperature (K)	80	80
Crystal system	orthorhombic	monoclinic
Space group	$P\ 2_1\ 2_1\ 2_1$	$P\ 2_1$
Unit cell lengths (Å)		
a	7.70	7.57
b	13.95	14.06
c	23.48	10.86
Unit cell angles (°)		
α	90.0	90.0
β	90.0	99.4
γ	90.0	90.0
Cell volume (Å^3)	2522.10	1140.39
No. of datasets merged	1	4
No. of observed reflections	7217	11064
No. of unique reflections	2051	1352
R_{obs} (%)	16.7	23.3
R_{meas} (%)	19.6	24.9
I/Sigma	6.53	7.24
$CC_{1/2}$	97.2	97.0
Completeness (%)	96.4	94.9
**Resolution (Å)**	**0.90**	**0.96**
**R_1 (%)**	**16.4**	**15.3**
wR_2 (%)	37.1	37.9
GooF	1.290	1.341

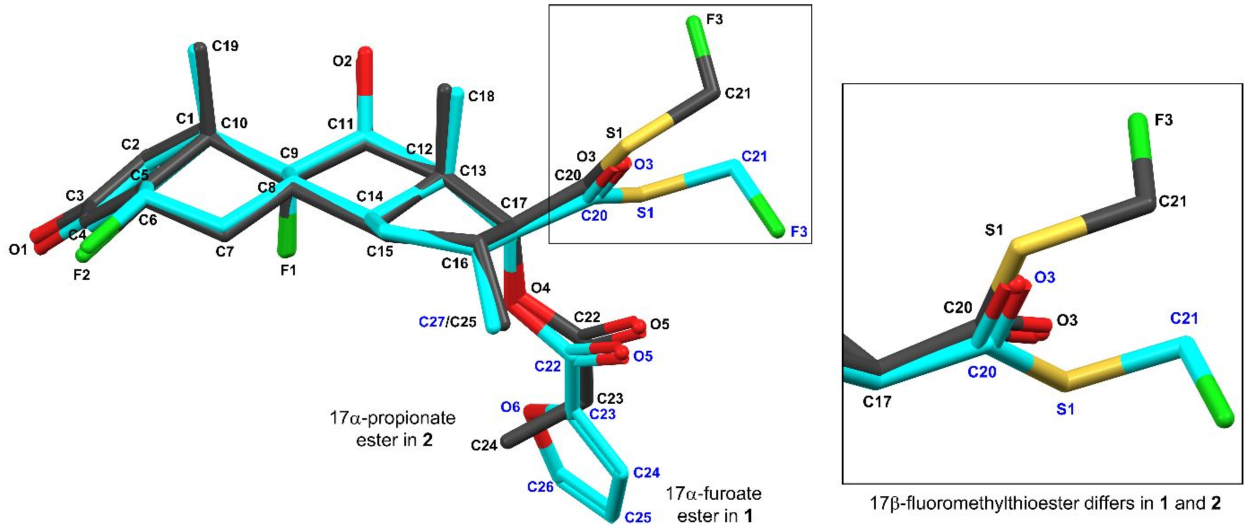

Figure S1 Overlay of the MicroED structures of **1** and **2** in solid state. Carbon atoms of **1** were colored in cyan, carbon atoms of **2** were colored in grey. Numbering of **1** was colored in blue, numbering of **2** was colored in black. Atoms with close locations shared same labels and were colored in black for clarification. 17β-substitutions shown the major conformational differences and were expanded. The minor conformation of **2** in solid state was omitted for clarification.

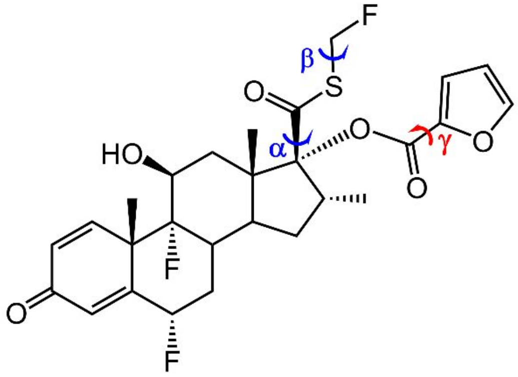

Fluticasone furoate 1

α: C13–C17–C20–S1 (set 1)
β: C20–S1–C21–F3 (set 2)
γ: O4–C22–C23–O6 (set 3)

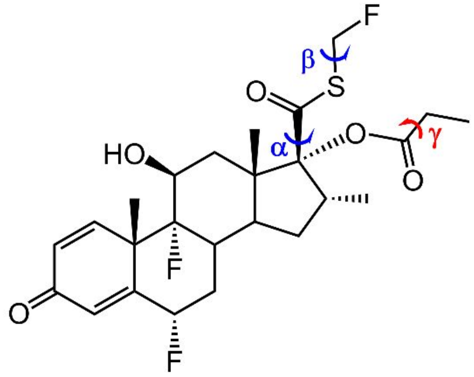

Fluticasone propionate 2

α: C13–C17–C20–S1 (set 1)
β: C20–S1–C21–F3 (set 2)
γ: O4–C22–C23–C24 (set 3)

Figure S2 Structure models and selected torsion angles used for DFT calculations. In sets 1-3 for **1**, α (C13–C17–C20–S1), β (C20–S1–C21–F3), or γ (O4–C22–C23–O6) was rotated and fixed from 0° to 360° with 15° increment, separately. The remaining structure was allowed to freely optimize. In sets 1-3 for **2** (the minor conformation was omitted), α (C13–C17–C20–S1), β (C20–S1–C21–F3), or γ (O4–C22–C23–C24) was rotated and fixed from 0° to 360° with 15° increment, separately. The remaining structure was allowed to freely optimize.

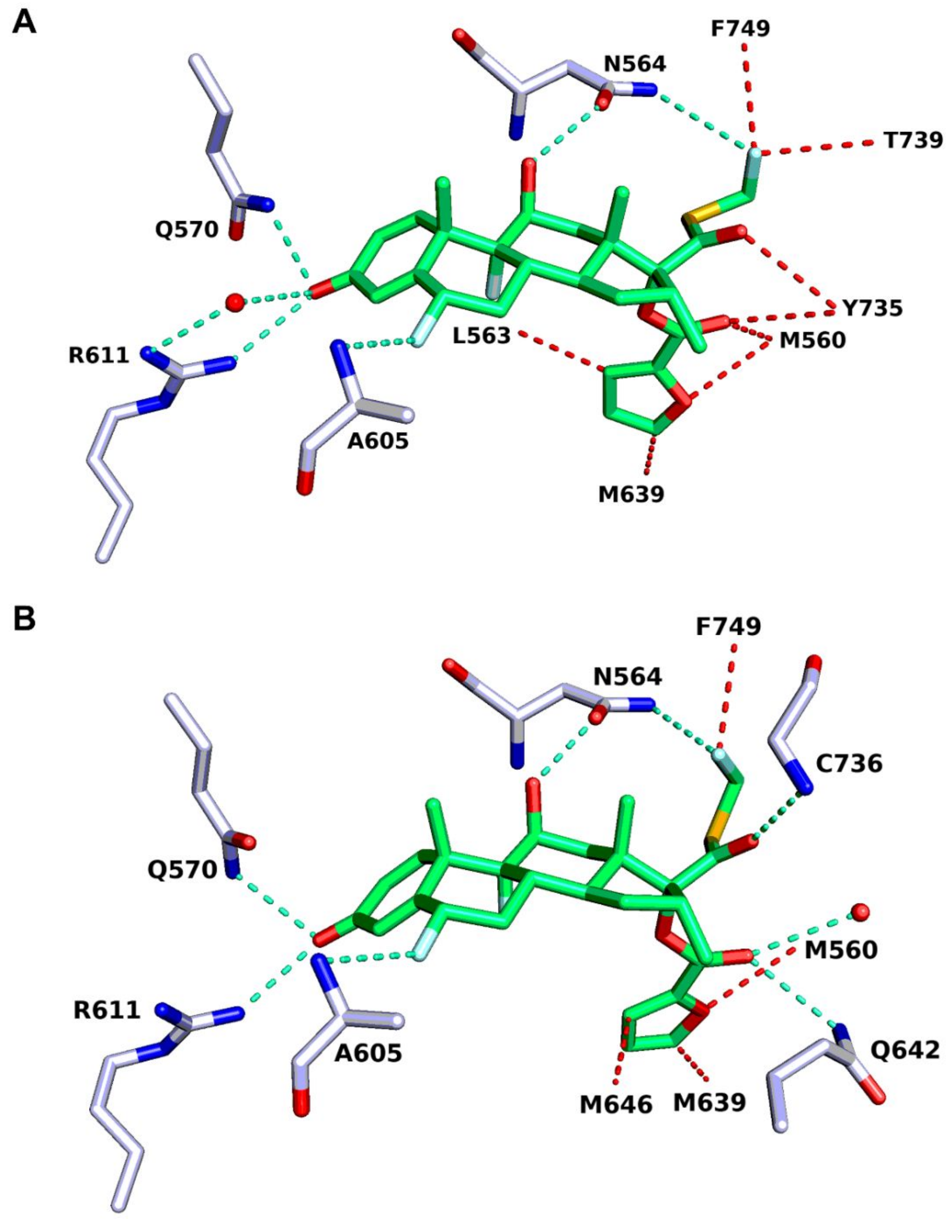

Figure S3 Interaction between **1** and residues in glucocorticoid receptor (GR). (A) PDB entry: 3CLD;[23] (B) PDB entry: 7PRV.[24] Hydrogen bonding were colored in dashed cyan lines, selected hydrophobic interactions were colored in dashed red lines.

Reference

1 Hattne, J.; Martynowycz, M. W.; Penczek, P. A.; Gonen, T. MicroED with the Falcon III direct electron detector. *IUCrJ* **2019**, *6* (5), 921-926.
2 Hattne, J.; Reyes, F. E.; Nannenga, B. L.; Shi, D.; De La Cruz, M. J.; Leslie, A. G. W.; Gonen, T. MicroED data collection and processing. *Acta Crystallographica Section A: Foundations and Advances* **2015**, *71* (4), 353-360.
3 Kabsch, W. xds. *Acta Crystallogr., Sect. D: Biol. Crystallogr.* **2010**, *66* (2), 125-132.
4 Kabsch, W. Integration, scaling, space-group assignment and post-refinement. *Acta Crystallogr., Sect. D: Biol. Crystallogr.* **2010**, *66* (2), 133-144.
5 Schneider, T. R.; Sheldrick, G. M. Substructure solution with SHELXD. *Acta Crystallogr., Sect. D: Biol. Crystallogr.* **2002**, *58* (10), 1772-1779.
6 Sheldrick, G. M. Crystal structure refinement with SHELXL. *Acta Crystallogr., Sect. C: Struct. Chem.* **2015**, *71* (1), 3-8.
7 Hübschle, C. B.; Sheldrick, G. M.; Dittrich, B. ShelXle: a Qt graphical user interface for SHELXL. *Journal of applied crystallography* **2011**, *44* (6), 1281-1284.
8 Neese, F. Software update: The ORCA program system—Version 5.0. *Wiley Interdiscip. Rev. Comput. Mol. Sci.* **2022**, *12* (5), e1606.
9 Tirado-Rives, J.; Jorgensen, W. L. Performance of B3LYP density functional methods for a large set of organic molecules. *J. Chem. Theory Comput.* **2008**, *4* (2), 297-306.
10 Petersson, a.; Bennett, A.; Tensfeldt, T. G.; Al-Laham, M. A.; Shirley, W. A.; Mantzaris, J. A complete basis set model chemistry. I. The total energies of closed-shell atoms and hydrides of the first-row elements. *J. Chem. Phys.* **1988**, *89* (4), 2193-2218.
11 Barone, V.; Cossi, M. Quantum calculation of molecular energies and energy gradients in solution by a conductor solvent model. *J. Phys. Chem. A* **1998**, *102* (11), 1995-2001.
12 Marenich, A. V.; Cramer, C. J.; Truhlar, D. G. Universal solvation model based on solute electron density and on a continuum model of the solvent defined by the bulk dielectric constant and atomic surface tensions. *J. Phys. Chem. B* **2009**, *113* (18), 6378-6396.
13 Chai, J.-D.; Head-Gordon, M. Systematic optimization of long-range corrected hybrid density functionals. *J. Chem. Phys.* **2008**, *128* (8).
14 McLean, A. D.; Chandler, G. S. Contracted Gaussian basis sets for molecular calculations. I. Second row atoms, Z= 11–18. *J. Chem. Phys.* **1980**, *72* (10), 5639-5648.
15 Prism, G. GraphPad Prism Version 8 for Windows. *GraphPad Software, San Diego, California, USA* **2018**.
16 Kovacsne-Mezei, A.; Gabriel, R.; Jegorov, A. Polymorphs of Fluticasone Furoate and Processes for Preparation Thereof. U.S. Patent US20100240629A1, September 23, **2010**.
17 Biggadike, K.; Coote, S. J.; Craig, A. S.; Jacewicz, V. W.; Millan, M. J.; Nice, R. K.; Noga, B. M.; Seager, J. F.; Theophilus, A. L.; Crowe, D. M. Anti-inflammatory Androstane Derivative Compositions. U.S. Patent US6777399B2, August 17, **2004**.
18 Biggadike, K.; Coote, S. J.; Craig, A.; Jacewicz, V.; Millan, M. J.; Seager, J. F.; Theophilus, A. L. Anti-inflammatory Androstane Derivative Compositions. U.S. Patent US6777400B2, August 17, **2004**.

19 Biggadike, K.; Chetina, O.; Coote, S. J.; Craig, A.; Jacewicz, V.; Millan, M. J.; Seager, J. F.; Theophilus, A. L. Anti-inflammatory Androstane Derivative Compositions. U.S. Patent US6858593B2, February 22, **2005**.
20 Coote, S. J.; Nice, R. K.; Wipperman, M. D. Process for the Production of Fluticasone Propionate, in Particular of Polymorphic Form 1. European Patent EP1474436B1, October 28, **2009**.
21 Cejka, J.; Kratochvil, B.; Jegorov, A. Crystal structure of fluticasone propionate, C25H31F3O5S. *Z. fur Krist. - New Cryst. Struct.* **2005**, *220* (2), 143-144.
22 Kariuki, B.; Harris, K. M.; Johnston, R.; Lancaster, R.; Staniforth, S.; Cooper, S. Structure determination of a steroid directly from powder diffraction data. *Chem. Commun.* **1999**, (17), 1677-1678.
23 Biggadike, K.; Bledsoe, R. K.; Hassell, A. M.; Kirk, B. E.; McLay, I. M.; Shewchuk, L. M.; Stewart, E. L. X-ray crystal structure of the novel enhanced-affinity glucocorticoid agonist fluticasone furoate in the glucocorticoid receptor−ligand binding domain. *J. Med. Chem.* **2008**, *51* (12), 3349-3352.
24 Postel, S.; Wissler, L.; Johansson, C. A.; Gunnarsson, A.; Gordon, E.; Collins, B.; Castaldo, M.; Köhler, C.; Öling, D.; Johansson, P. Quaternary glucocorticoid receptor structure highlights allosteric interdomain communication. *Nat. Struct. Mol. Biol.* **2023**, *30* (3), 286-295.
