# Supplementary material for: MicroED Structures of Fluticasone Furoate and Fluticasone Propionate Provide New Insights into Their Function
Source: Cryst Growth Des. 2025 Feb 12;25(5):1588–96. doi: 10.1021/acs.cgd.4c01683 (PMC11887049; doi:10.1021/acs.cgd.4c01683)
Supplement: Supplementary file 2 — cg4c01683_si_002.pdf [file cg4c01683_si_002.pdf]

For Table of Contents Use Only

MicroED Structures of Fluticasone Furoate and Fluticasone Propionate Provide New Insights to Their Function

Jieye Lin[1], Johan Unge[2] and Tamir Gonen[1,3,4]*

[1] Department of Biological Chemistry, University of California, Los Angeles, 615 Charles E. Young Drive South, Los Angeles, California 90095, United States

[2] Department of Chemistry, Umeå University, 901 87 Umeå, Sweden

[3] Department of Physiology, University of California, Los Angeles, 615 Charles E. Young Drive South, Los Angeles, California 90095, United States

[4] Howard Hughes Medical Institute, University of California, Los Angeles, Los Angeles, California 90095, United States

* Corresponding Author T.G. tgonen@g.ucla.edu

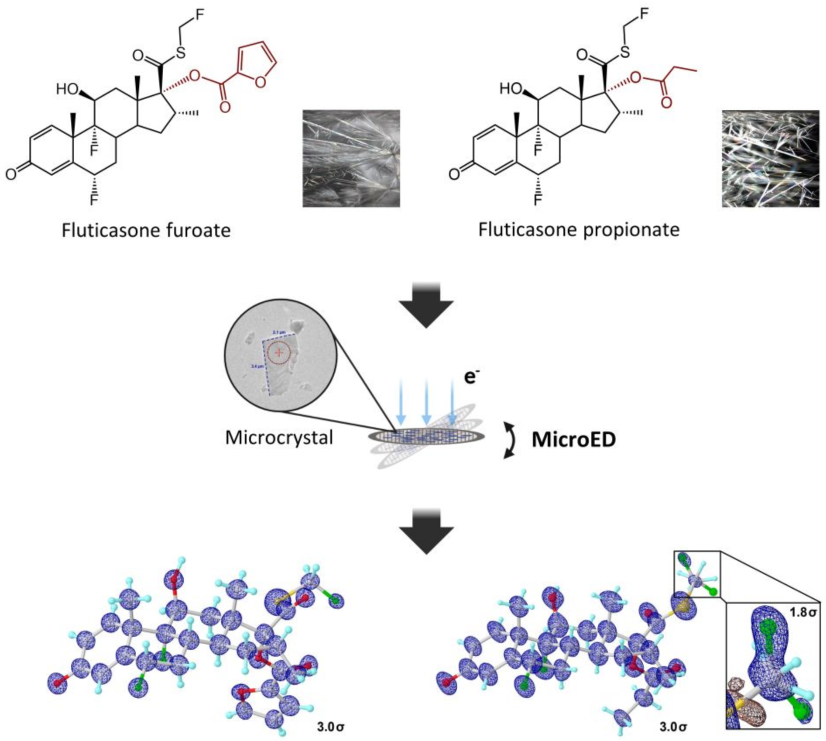

Synopsis: The 3D structures of fluticasone furoate **1** and fluticasone propionate **2** in solid states were solved by microcrystal electron diffraction (MicroED). A comparative analysis of structures of **1** and **2** across three states (in solid state, in solution, and protein bound conformation) revealed the conformational and energy changes during drug administration.
